# Short diameter may be a useful simple indicator of the tumor response in skull base meningiomas after conventionally fractionated stereotactic radiotherapy

**DOI:** 10.1007/s00330-021-07707-1

**Published:** 2021-02-10

**Authors:** Keiichi Takehana, Daisuke Nakamura, Alshaymaa Abdelghaffar, Megumi Uto, Tomohiro Katagiri, Yoshiki Arakawa, Yohei Mineharu, Susumu Miyamoto, Takashi Mizowaki

**Affiliations:** 1 Department of Radiation Oncology and Image-Applied Therapy, Kyoto University Graduate School of Medicine, 54 Shogoin Kawahara-cho, Sakyo-ku, Kyoto, 606-8507 Japan; 2 Department of Radiological Sciences, Graduate School of Biomedical Sciences, Nagasaki University, Nagasaki, Japan; 3 Department of Clinical Oncology, Sohag University Hospital, Sohag University, Sohag, Egypt; 4 Department of Neurosurgery, Kyoto University Graduate School of Medicine, Kyoto, Japan

**Keywords:** Meningioma, Stereotactic radiation therapy, Follow-up studies, Skull base, Response Evaluation Criteria in Solid Tumors

## Abstract

**Objectives:**

The purpose of this study was to assess the radiological change patterns in skull base meningiomas after conventionally fractionated stereotactic radiotherapy (CFSRT) to determine a simple and valid method to assess the tumor response.

**Materials and methods:**

Forty-one patients with a benign skull base meningioma treated by CFSRT from March 2007 to August 2015 were retrospectively evaluated. We measured tumor volume (TV), long-axis diameter (LD), and short-axis diameter (SD) on both pre-treatment images and follow-up images of 1, 3, and 5 years after CFSRT, respectively. The paired *t* test was used to detect differences in the LD and SD change rates. Spearman’s correlation coefficients were calculated to evaluate relationships between the TV and the diameters changes.

**Results:**

The number of available follow-up MRIs that was performed at 1, 3, and 5 years after the CFSRT was 41 (100%), 34 (83%), and 23 (56%), respectively. The change rates of SD were significantly higher than those of LD at every time point and more strongly correlated with the change rates of tumor volume at 3 and 5 years after CFSRT.

**Conclusions:**

SD may be useful as a simple indicator of the tumor response for skull base meningioma after CFSRT.

**Key Points:**

*• The change rate in short-axis diameter is a useful and simple indicator of the response of skull base meningioma to conventionally fractionated stereotactic radiotherapy.*

*• Conventionally fractionated stereotactic radiotherapy for skull base meningioma achieved excellent 5-year local control.*

**Supplementary Information:**

The online version contains supplementary material available at 10.1007/s00330-021-07707-1.

## Introduction

A meningioma is the most common primary intracranial tumor, and its annual incidence is 6 per 1 million people [1]. Although 70–80% of meningiomas are benign, they can be severely disabling and life threatening depending on their location [2]. Managing a skull base meningioma is challenging because of the existence of critical vascular structures and cranial nerves adjacent to or within the tumors. Radical complete resection for a skull base meningioma is often difficult and may cause serious morbidity. Consequently, a high frequency of local recurrence has been reported after surgery alone regardless of the pathological type [3, 4].

Radiotherapy has an important role in the management of a skull base meningioma that is unresectable or for residual tumors after surgery [5].

Follow-up after conventionally fractionated stereotactic radiotherapy (CFSRT) consists of regular outpatient visits and image analyses. However, the evaluation criteria of the tumor response widely varied in previous reports, such as a tumor diameter or volume. These differences in the response assessment make it difficult to compare one study to another [6–9].

Volumetric assessments are the most precise method for evaluating the tumor response, but they are time consuming to perform in daily clinical practice. According to the Response Evaluation Criteria in Solid Tumors (RECIST) criteria, long-axis diameter (LD) is used to assess tumors. In clinical practice, we have sometimes experienced cases of a skull base meningioma treated by radiotherapy, whose LD often remained stable on follow-up images, while SD had apparently decreased in size. However, to the best of our knowledge, no study has investigated whether SD is an appropriate measurement approach for evaluating tumor response for skull base meningiomas after CFSRT. Therefore, we aimed to evaluate the radiological pattern of change focusing on SD changes in skull base meningiomas after CFSRT.

## Materials and methods

All procedures performed in studies involving human participants were in accordance with the ethical standards of the institutional and/or national research committee and with the 1964 Declaration of Helsinki and its later amendments or comparable ethical standards. This data analysis was approved by the Ethics Committee of Kyoto University Hospital (approval number: E-2277). We obtained written informed consent for the treatment and the research use of the clinical data from each patient.

### Patient characteristics

Forty-nine consecutive patients with a skull base meningioma received radiotherapy at our hospital from February 2007 to August 2015. Eight patients were excluded from analyses for the following reasons: (1) received hypofractionated stereotactic radiotherapy, (2) previous radiotherapy at the same site, and (3) atypical or anaplastic meningioma. Data from the remaining 41 patients were retrospectively analyzed in this study.

### Radiotherapy

Patients were immobilized in a thermoplastic flat shell (Klarity) with infrared markers for image guidance. Contrast-enhanced computed tomography (CT) images were acquired with a Light Speed RT instrument (GE Healthcare) at a slice thickness of 1.25 mm. Treatment plans were developed with BrainSCAN version 5.3.1 (BrainLAB), iPlan RT Dose version 4.5.1 (BrainLAB), or Eclipse version 8.6 (Varian Medical Systems). Contrast-enhanced CT images were fused with contrast-enhanced magnetic resonance imaging (MRI) images on a radiotherapy planning system.

Thirty-five patients underwent radiotherapy using the Novalis system with 6-MV linear accelerator, m3 micro-multileaf collimator, the ExacTrac X-ray system, and the Robotic Tilt Motion mounted on the Exact Couch top (BrainLAB). Five patients were treated with the Vero4DRT (Hitachi, Ltd.), and one patient was treated with the Clinac iX (Varian Medical Systems).

Gross tumor volume (GTV) was defined as the volume of the tumor on contrast-enhanced T1-weighted MRI. The clinical target volume (CTV) was defined as the GTV with a thickened dural tail. The planning target volume (PTV) was defined as the CTV plus a 1–2-mm margin in all directions. CFSRT was performed using multiple dynamic conformal arc therapy (DCAT) or by intensity-modulated radiotherapy (IMRT). The PTV was covered by 90% of the prescribed dose in both the DCAT and IMRT plans.

### Radiological evaluation

**Fig. 1:**
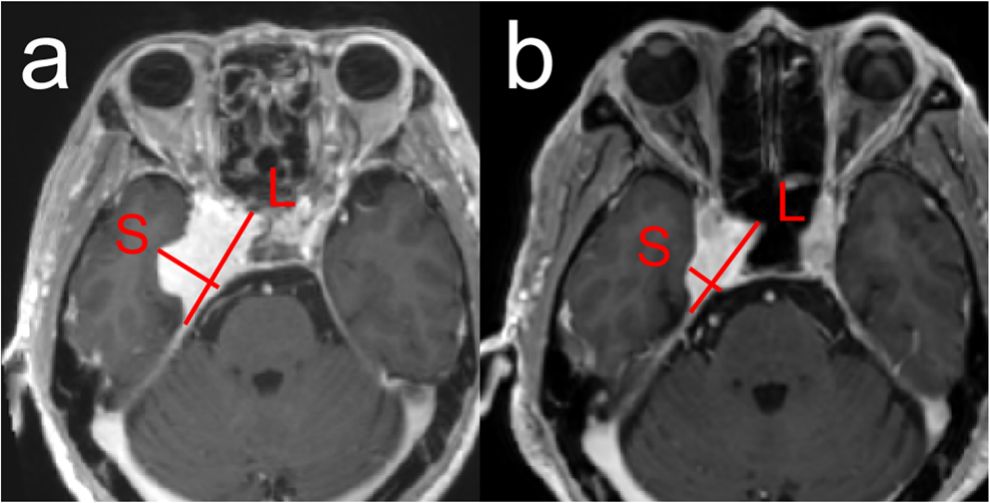
Radiographic change in a representative case. **a** Contrast-enhanced T1-weighted magnetic resonance imaging (MRI) image of a cavernous sinus meningioma before conventionally fractionated stereotactic radiotherapy (CFSRT). **b** Contrast-enhanced T1-weighted MRI 35 months after CFSRT. L, long-axis diameter of the tumor; S, short-axis diameter of the tumor

The tumor volume (TV), LD, and short-axis diameter (SD) of the tumors were evaluated by MRI at the beginning of CFSRT and at 1, 3, and 5 years after the CFSRT. TV, LD, and LD were measured on the MIM (MIM Software Inc.). All images were transferred from PACS to the MIM, then the patient-specific information was removed, and a unique research ID was assigned to each patient before the measurement sessions. We selected an image, where the tumor diameter was longest, from axial, coronal, and sagittal planes on the pre-treatment MRI. Then, LD was measured as the longest diameter on the selected image, and SD was defined as the longest tumor diameter in the direction perpendicular to the LD on the same image (Fig. 1). Post-treatment MRIs were registered with pre-treatment MRI using a rigid registration algorithm implemented in the MIM. On the post-treatment MRIs, LDs and SDs were measured at the corresponding locations on the corresponding images to the pre-treatment measurements. The LDs and SDs were measured by a radiation oncologist (K.T.) with 9 years of experience and a radiation oncologist (A.A.) with 11 years of experience, independently. The two readers each performed two measurements with washout periods of at least 1 month between measurements. TV was measured by manual segmentation by K.T. using MIM (MIM Software Inc.) after the second tumor diameter measurement session. Intra-class correlation coefficients (ICCs) were calculated to evaluate intra- and inter-observer agreement in terms of the LD and SD values. The values used in analyses were calculated by averaging the four measurements made by the two readers for each tumor.

Tumor response was judged based on the TV at the last imaging follow-up. A 50% or more reduction in the TV was defined as a partial response (PR). A 10% or more increase in the TV was defined as progressive disease (PD). No decrease to a PR or an increase to a PD was defined as no change (NC).

### Follow-up and statistical analyses

Patients were routinely followed up every 3–6 months for the first 3 years and then annually. Overall survival (OS) was calculated from the start of radiotherapy to death from any cause or censored at the last follow-up. Local control (LC) was calculated from the start of radiotherapy to PD or censored at the last follow-up or death from another cause. Toxicities were evaluated using the Common Terminology Criteria for Adverse Events, version 4. Acute radiation toxicities are side effects that occur after CFSRT or in the 3 months after the end of treatment. Late toxicity was defined as a complication occurring 3 months after the end of CFSRT.

All statistics were calculated using R version 3.32 (The R language for Statistical Computing). OS and LC were estimated using the Kaplan–Meier method. The paired *t* test was used to detect differences in the LD and SD change rates. Spearman’s correlation coefficients (ρ) were calculated to evaluate relationships between the TV change rates and the diameters. A *p* value < 0.05 was considered significant.

## Results

### Clinical characteristics and treatment parameters

The patient characteristics are shown in Table 1. Among 26 patients who received RT after surgery, nine patients received RT immediately after surgery and 17 patients received RT when the tumor size was shown to have increased on follow-up images. The median time from surgery to CFSRT was 17.6 months (range: 1–226 months). The median prescribed dose was 52.2 Gy (range: 46.8–54.0 Gy), which was delivered in a daily fraction size of 1.8 Gy (26–30 fractions). The median PTV was 21.5 cm^3^ (range: 3.4–98.3 cm^3^). Thirty-four patients were treated by DCAT, and seven patients were treated by IMRT.

**Table 1:** Patient characteristics

Characteristic		*N*
Sex	Male/female	15/26
Age	Median (range)	59 (11–83)
Location	Cavernous sinus Clivus/petroclival Jugular foramen Optic nerve sheath Tuberculum sellae Sphenoid ridge Olfactory groove Posterior fossa	14 12 5 3 3 2 1 1
Prior surgery	Yes/no	26/15
Simpson’s grade	I/II/III/IV/unknown	0/1/0/23/2

### Tumor response assessment

For the LD and SD measurements, ICCs for intra- and inter-observer variability were higher than 0.96 (range, 0.96–0.99). The detailed data are provided in the supplementary material (supp. Table 1).

**Fig. 2:**
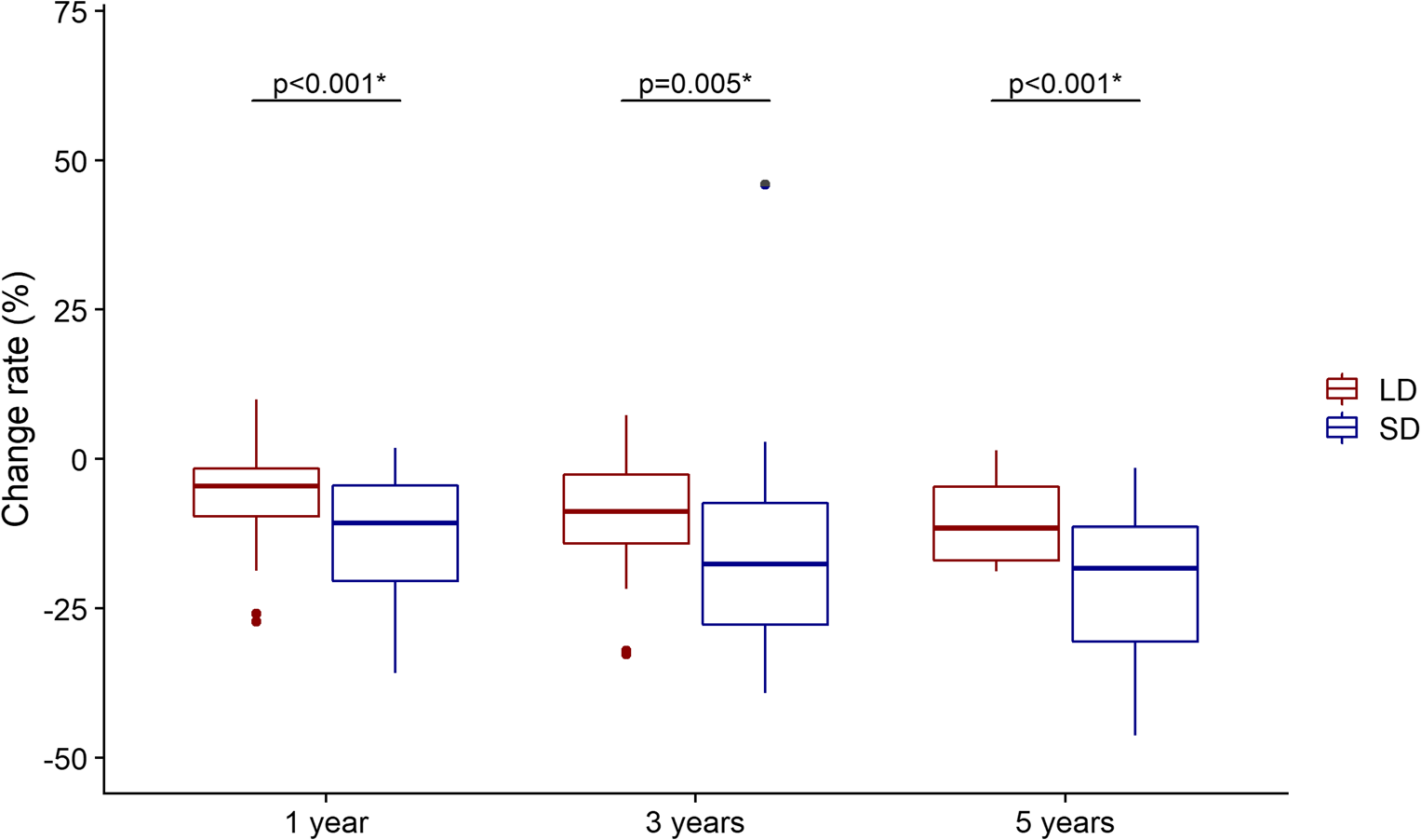
A comparison of change rates in the long- and short-axis diameters on pre-treatment and follow-up magnetic resonance imaging

**Fig. 3:**
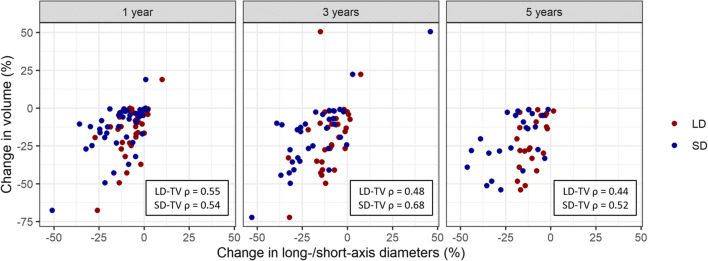
A scatterplot of the rates of change in tumor volume and long-/short-axis diameters

The number of available follow-up MRIs that was performed at 1, 3, and 5 years after the CFSRT was 41 (100%), 34 (83%), and 23 (56%), respectively. The mean slice thickness was 1.9 mm (range: 0.7–6.0), and the mean interslice gap was 0.2 mm (range: 0–1.5). The mean values and change rates of the LD, SD, and TV on pre-treatment and post-treatment MRIs were shown in Table 2. The change rate in the SD was significantly greater than that of the LD at 1 year (- 13% vs. - 6%, *p* < 0.001), 3 years (- 17% vs. - 9%, *p* = 0.005), and 5 years (- 21% vs. - 10%, *p* < 0.001) (Fig. 2). The change rates of the SD were more strongly correlated with those of the TV compared to those of the LD that were higher at 3 years (LD-TV: *ρ* = 0.48 vs. SD-TV: *ρ =* 0.68) and 5 years (LD-TV: *ρ* = 0.43 vs. SD-TV: *ρ* = 0.52) (Fig. 3). In 34 (83%) cases, the LD corresponded to the dural attachment of the skull base, except for 3 (7%) optic nerve sheath meningioma and 4 (10%) tumors which protrude through the skull foramen.

**Table 2:** The statistics of long-/short-axis diameters and tumor volume

	Pre-treatment MRI (*N* = 41)	1 year (*N* = 41)	3 years (*N* = 34)	5 years (*N* = 23)
LD (mm), mean (range)	34 (13–61)	32 (11–62)	31 (11–60)	29 (19–61)
LD change (%), mean (range)	–	− 6 (− 27–10)	− 9 (− 33–7)	− 10 (− 19–1)
SD (mm), mean (range)	21 (7–52)	18 (5–47)	17 (4–42)	17 (6–41)
SD change (%), mean (range)	–	− 13 (− 51–2)	− 17 (− 53–46)	− 21 (− 46 to –1)
Volume (cc), mean (range)	10.9 (1–61)	9.91 (1–60)	8.8 (1–38)	8.9 (1–38)
Volume change (%), mean (range)	–	–13 (− 68–19)	− 18 (− 72–50)	− 22 (− 54 to − 1)

*LD* long-axis diameter, *SD* short-axis diameter

### Overall survival and local control rate

The median overall treatment period was 41 days (range: 37–51 days). The median follow-up period was 64 months (range: 18–119 months). The 5-year OS and LC were 96.8% (95% confidence interval [CI]; 90.8–100) and 89.9 (95% CI; 79.5–100), respectively. Seven, 30, and 4 patients were judged as PR, NC, and PD at the last imaging follow-up, respectively.

### Neurological symptoms

Symptoms due to the tumor were present in 35 patients at the beginning of CFSRT, and the remaining 6 patients were asymptomatic. The symptoms improved in 13 (31.7%) patients after CFSRT. Diplopia or ptosis improved in six patients, facial numbness improved in four, low vision improved in one, and an olfactory disorder improved in one. One patient had an exacerbation of trigeminal neuralgia, and another patient had an exacerbation of ptosis after CFSRT. The symptoms in 20 patients remained unchanged.

The 35 symptomatic patients were divided into two groups, the improved group (*n* = 13) and the non-improved group (*n *= 22). The change rates in the tumor volume of the improved group were significantly greater than those in the non-improved group at 1 year after CFSRT (− 16% vs. − 6%, *p* = 0.03). On the other hand, the change rate in LD or SD was not statistically significant between the groups, although the change rates in SD of the improved group were greater than those in the non-improved group (− 15% vs. − 10%, *p *= 0.19). The detailed data are provided in the supplementary material (supp. Fig. 1).

### Treatment toxicities

Clinically significant acute adverse events developed in nine patients (22.0%), such as nausea, alopecia, and dizziness, but no grade 3 or higher acute adverse event was observed.

Late adverse events developed in five patients (12.2%). An exacerbation of neurological symptoms was observed in three patients, two patients developed trigeminal neuralgia, and one had ptosis without tumor progression. A growth disturbance was observed in an 11-year-old patient with a cavernous sinus meningioma, although no apparent hormone abnormality was detected after CFSRT. One patient died due to a tumor hemorrhage 40 months after CFSRT.

## Discussion

We demonstrated that the change rates in the SD were significantly greater than those in the LD in patients with skull base meningioma treated by CFSRT: − 13% vs. − 6% (*p* < 0.001) at 1 year, − 17% vs. − 9% (*p* = 0.005) at 3 years, and − 21% vs. − 10% (*p <* 0.001) at 5 years, respectively. We also demonstrated that the change rates in the SD were more strongly correlated with those of the TV if compared to the change rates in the LD at 3 years (LD-TV: *ρ* = 0.48 vs. SD-TV: *ρ* = 0.68) and 5 years (LD-TV: *ρ* = 0.43 vs. SD-TV: *ρ* = 0.52).

There is no clear consensus regarding the response criteria for meningiomas after radiotherapy [10]. Some groups have evaluated the tumor response using the change in TV, and other groups use the change in LD (Table 3) [6–9].

**Table 3:** Literature review of the tumor response criteria for skull base meningiomas

Author (year)	Scale	Criteria	Response at the last imaging follow-up
Uy (2002) [8]	Volume	PR: At least a 25% decrease	9 (24.3%)
		NC: Neither a decrease nor an increase	27 (73.0%)
		PD: At least a 25% increase	1 (2.7%)
Selch MT (2004) [9]	Long-axis diameter	CR: Tumor absence	0 (0%)
		PR: At least a 50% decrease	8 (17.8%)
		NC: Less than a 50% decrease	36 (80.0%)
		PD: Any increase	1 (2.2%)
Morimoto M (2011) [10]	Long-axis diameter	PR: At least a 50% decrease	3 (9.4%)
		NC: Neither a decrease nor an increase	25 (78.1%)
		PD: At least a 50% increase	4 (12.5%)
Navarria P (2015) [11]	Volume	PR: Any decrease	9 (36.0%)
		NC: No change	16 (64.0%)
		PD: At least a 10% increase	0 (0%)
Present study	Volume	PR: At least a 50% decrease	7 (17.1%)
		NC: Neither a decrease nor an increase	30 (73.2%)
		PD: At least a 10% increase	4 (9.8%)

*PR* partial response, *NC* no change, *PD* progressive disease, *CR* complete response

We evaluated the change rate in the SD, which reflected the change rate in the TV compared to that in the LD after CFSRT. Most of the LD coincides with dural attachment in cases of meningioma and remains almost unchanged due to dural fixation. The change in the LD was unsuitable as an index for detecting the tumor response. Although the LD of tumor lesions, except lymph node metastases, should be evaluated according to the RECIST criteria, the mean change rate in the LD was only − 10% at 5 years after CFSRT in this study, and almost all tumors remained within the range of stable disease [11]. According to the Response Assessment in RANO Criteria, the tumor response is partly defined by the product of the LD and SD of a contrast-enhanced lesion [12], and the LD seems to contribute little to this assessment compared to its contribution to that of other intracranial tumors, such as glioma.

On the other hand, most of the SD was measured in the direction from which the tumor expanded perpendicular to the skull base. The change rate in the SD was significantly greater than that of the LD and reflected the change rate in the TV. This is the first report to indicate the relationship between the change rate of the SD and TV for skull base meningioma after radiotherapy. The radiographic change in the SD was an effective and simple indicator of the tumor response.

Harrison et al assessed the time course of volume changes in 252 meningiomas treated with stereotactic radiosurgery and found that transient enlargement occurred in 15 (6.0%) tumors, and transient regression occurred in 6 (2.4%) [13]. Although these transient changes may have affected the results, they suggested that such changes tend to occur in meningiomas during the first 3–6 months after treatment. As we evaluated the patients who were followed for at least 1 year, the transient changes are unlikely to have had a significant impact on the results of this study.

Definitive CFSRT for a meningioma achieves excellent local control. The 5-year OS and LC after fractionated radiotherapy have been reported to be 93–97% and 89–96%, respectively [5, 6]. Although late-onset recurrence of a meningioma after radiotherapy is not rare because of its slow-growing nature, studies that have reported long-term outcomes with > 5 years of median follow-up period are scarce [14–18]. We demonstrated similar clinical outcomes with our series and a relatively long follow-up period.

As described above, most of the LD coincided with dural attachment, and the SD was measured in the direction in which the tumor expanded perpendicular from the skull base. Thus, shrinkage of the SD may reflect a relief of compression to the cranial nerves or a normal brain even if the volume change appeared to be relatively small. In this study, the change rates in SD of the imlimitations of our study were its retrospective nature,proved group were greater than those of the non-improved group, although the difference was not statistically significant (− 15% vs. − 10%, *p *= 0.19). Further study with an increased number of patients is needed to clarify the relationship between improvements in symptoms and the pattern of tumor shrinkage.

The limitations of our study were its retrospective nature, its relatively small sample size, and that about one-third of the patients were treated without a histological diagnosis. Variability in slice thickness and the gadolinium dose could have caused measurement and image registration errors. To the best of our knowledge, there has been no study purposely validating the registration accuracy of the rigid registration in non-deforming regions such as skull base. However, the overall target localization error of a non-invasive stereotactic system, based on the target localization using a rigid image registration data of the skull, was reported as ±0.6 mm in each direction in the phantom study [19]. Because the error originating from the image registration is considered to be only a fraction of the reported overall error, the report indirectly suggests the rigid registration of the skull is highly accurate. Analyses regarding prognostic or predictive factors for LC, OS, or treatment toxicities did not get executed because only four patients have recurred, and one patient had died at the last follow-up. In addition, further evaluation is mandatory to confirm whether this result can be applicable to meningiomas located in other locations or treated with different types of radiation, because the sample size was limited due to the rarity of the disease and the single-institution study.

Our study demonstrated that the radiological change in the SD was more strongly correlated than that in the LD with the change in the TV and achieved excellent LC. This is the first report about a change in the SD after radiotherapy of a skull base meningioma. The change rate in the SD was useful as a simple indicator of the response to CFSRT.

## Supplementary information

ESM 1 (DOCX 269 kb)

ESM 2 (DOC 87 kb)

## Abbreviations

CFSRT: Conventionally fractionated stereotactic radiotherapy
CTV: Clinical target volume
DCAT: Dynamic conformal arc therapy
GTV: Gross tumor volume
IMRT: Intensity-modulated radiotherapy
LC: Local control
LD: Long-axis diameter
NC: No change
OS: Overall survival
PD: Progressive disease
PR: Partial response
PTV: Planning target volume
SD: Short-axis diameter
TV: Tumor volume
