# Supplementary material for: Short diameter may be a useful simple indicator of the tumor response in skull base meningiomas after conventionally fractionated stereotactic radiotherapy
Source: Eur Radiol. 2021 Feb 10;31(8):6367–73. doi: 10.1007/s00330-021-07707-1 (PMC8270820; doi:10.1007/s00330-021-07707-1)

**Supplementary document**

Table 1. Intra-class correlation coefficients of the inter- and intra-observer variabilities in terms of long- and short-axis diameter measurements.

|  | Intra-observer variability | | | | Inter-observer variability | |
| --- | --- | --- | --- | --- | --- | --- |
|  | Reader 1 | | Reader 2 | | LD | SD |
|  | LD | SD | LD | SD |  |  |
| Pre-treatment MRI | 0.96 | 0.97 | 0.99 | 0.98 | 0.98 | 0.98 |
| 1 year | 0.97 | 0.98 | 0.99 | 0.98 | 0.98 | 0.98 |
| 3 years | 0.97 | 0.97 | 0.98 | 0.98 | 0.98 | 0.97 |
| 5 years | 0.98 | 0.98 | 0.97 | 0.98 | 0.97 | 0.97 |

LD – long-axis diameter, SD – short-axis diameter

Table 2. Spearman and Pearson’s correlation coefficients between long/short-axis diameter and tumor volume after conventionally fractionated stereotactic radiotherapy.

|  | **LD-TV** | **SD-TV** | **p value** |
| --- | --- | --- | --- |
| Pearson’s r  1 year  3 years  5 years | 0.62  0.50  0.51 | 0.58  0.78  0.54 | 0.76  0.02*  0.88 |
| Spearman’s ρ  1 year  3 years  5 years | 0.55  0.48  0.44 | 0.54  0.68  0.52 |  |

LD - Long-axis diameter, SD - Short-axis diameter, TV- Tumor volume

P-values were calculated by z transformation using cocor package in R.

Figure 1. The comparison of the change rate in the long-/short-axis diameters and volume at one year after conventionally fractionated stereotactic radiotherapy between neurological symptom improve group and non-improved group.


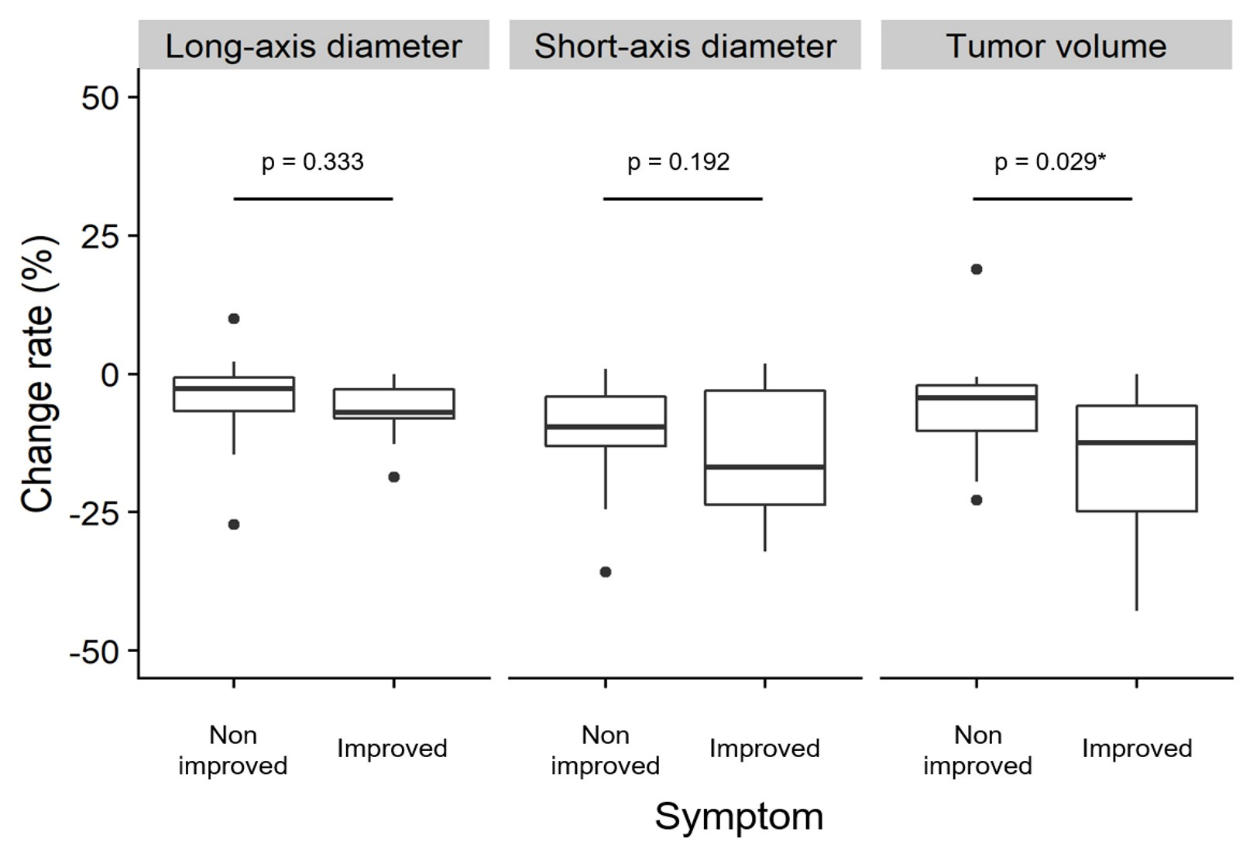

Supplement: Supplementary file 1 — (DOCX 269 kb) [file 330_2021_7707_MOESM1_ESM.docx]
